# Using Large Language Models to Extract and Assess Mobility Documentation for Age-Friendly Health Systems

**DOI:** 10.1093/geroni/igaf122.1184

**Published:** 2025-12-31

**Authors:** Huai Cheng, Betsy Yang, Anders Westanmo, Amy Gravely, Deborah Kado, Howard Fink

**Affiliations:** Minneapolis VA Hospital, Minneapolis, Minnesota, United States; GRECC at Palo Alto VA Health System, Palo Alto, California, United States; Minneapolis VA Health System, Minneapolis, Minnesota, United States; Research Service at Minneapolis VA Health Care System, Minneapolis, Minnesota, United States; GRECC at Palo Alto VA Health System, Palo Alto, California, United States; Minneapolis VA Health Care System, Minneapolis, Minnesota, United States

## Abstract

The Age-Friendly Health System (AFHS) uses the geriatric 4Ms framework (mind, mobility, medications, matters most) to improve health outcomes in older adults. Achieving AFHS Level 2 recognition in outpatient settings requires documentation of annual 4Ms care, but current methods to assess documentation, manual chart reviews or template from electronic health records are time-consuming and prone to inconsistency and errors. This study evaluates whether Large Language Models (LLMs) can accurately extract mobility-related documentation from patient charts, comparing their performance to manual reviews conducted by two geriatricians. Mobility documentation includes seven key components, including falls, activities of daily living (ADLs), instrumental ADLs, fall related injuries, gait and balance assessments, assistive device, and home safety. First, two geriatricians independently reviewed 26 structured geriatric consult notes (with a 4M-structured template) and 78 unstructured general medicine notes and reached consensus on mobility documentation. The LLMs prompts were fine-tuned to extract the same information from the structured notes. Next, we tested the prompts on the same 26 structured notes pooled with the 78 unstructured general medicine notes. We then assessed consensus on mobility documentation in these 104 notes between the two geriatricians (Cohen’s kappa 0.82), and geriatrician vs. LLMs (Cohen’s kappa 0.65), respectively, indicating moderate to substantial agreement. Our findings show that LLMs demonstrated high agreement with geriatricians across both note types, suggesting that LLMs can reliably chart review for mobility documentation in AFHS. This automation has the potential to streamline chart reviews, reduce documentation burden, enhance documentation compliance, and support the broader adoption of AFHS.

